# Validation Study of a New Random-Access Chemiluminescence Immunoassay Analyzer i-TRACK10^®^ to Monitor Infliximab and Adalimumab Serum trough Levels and Anti-Drug Antibodies

**DOI:** 10.3390/ijms23179561

**Published:** 2022-08-24

**Authors:** Anne Emmanuelle Berger, Aude Gleizes, Louis Waeckel, Xavier Roblin, Roman Krzysiek, Salima Hacein-Bey-Abina, Alessandra Soriano, Stephane Paul

**Affiliations:** 1 CIRI—Centre International de Recherche en Infectiologie, Team GIMAP (Saint-Etienne), Université Claude Bernard Lyon 1, Inserm, U1111, CNRS, UMR5308, ENS Lyon, UJM, F69007 Lyon, France; 2 CIC Inserm 1408 Vaccinology, F42023 Saint-Etienne, France; 3 Department of Immunology, iBIOTHERA Reference Center, University Hospital of Saint-Etienne, F42023 Saint-Etienne, France; 4 Immunology Laboratory, Groupe Hospitalier Universitaire Paris-Saclay, Hôpital Bicêtre, Assistance Publique-Hôpitaux de Paris, F94270 Paris, France; 5 Unité des Technologies Chimiques et Biologiques pour la Santé, Université de Paris, CNRS, INSERM, UTCBS, F75270 Paris, France; 6 Faculty of Pharmacy, Paris-Saclay University, F92290 Paris, France; 7 Gastroenterology Department, CHU Saint-Etienne, F42023 Saint-Etienne, France; 8 Faculté de Médecine, Université Paris-Saclay, UMR-996 INSERM Inflammation, Microbiome and Immunosurveillance, F92141 Clamart, France; 9 Gastroenterology Division and IBD Center, Internal Medicine Department, Azienda Unità Sanitaria Locale—IRCCS, 42122 Reggio Emilia, Italy

**Keywords:** TNF inhibitors, adalimumab, infliximab, therapeutic drug monitoring, anti-drug antibodies, CLIA, ELISA

## Abstract

**Background**. Monitoring of biological TNF inhibitors is a very important tool to guide clinical decisions using specialized algorithms, especially in gastroenterology. A new chemiluminescent instrument (i-TRACK^10®^ from Theradiag) could replace ELISA techniques to calculate the dosage of drugs and anti-drug antibodies. In this bi-centric study, we explored the analytical performances of i-TRACK^10®^ using manual or automated (DS2^®^) ELISA Lisa-Tracker^®^ assays, and compared the results. **Patients and methods**. Intra- and inter-run performances were evaluated with i-TRACK^10®^ in two different laboratories and for two different ranges of values for infliximab, adalimumab, and their respective antibodies. Patients’ samples were used in the labs to compare the results obtained between the new instrument and either the manual Lisa-Tracker^®^ or the automated DS2. **Results**. Intra- and inter-run performances were satisfactory, with values between 1.8% and 16.1% (for inter-run imprecision at low/medium values of infliximab). Results were generally comparable between assays. with the lowest value of correlation at 0.59 (anti-adalimumab dosage between i-TRACK^10®^ and manual ELISA). Most often, values of drugs and anti-drug antibodies were higher with i-TRACK^10®^ than with manual ELISA assay, and correlation values were better with automated ELISA. Agreements were globally acceptable, and the lowest coefficients of 0.7 was obtained for adalimumab values between i-TRACK^10®^ and the two ELISA methods, and for anti-adalimumab values between i-TRACK^10®^ and manual ELISA. The type of assay can potentially induce a change in the class of patients and lead to divergent therapeutic decisions. **Conclusions**. The new random-access instrument i-TRACK^10®^ presents many advantages in a routine laboratory: rapidity, the possibility of standardization, usability, and expansion of the measurement range. Despite the relatively good agreement of results, it is preferable to use the same assay in longitudinal follow-up of a patient, because quantitative results were not completely equivalent especially for anti-drug antibodies.

## 1. Background

Tumor necrosis factor (TNF) is recognized as a key player in a broad range of immune inflammatory diseases. The advent of TNF inhibitors (TNFi) has progressively changed the therapeutic field, notably for inflammatory bowel and rheumatologic diseases [1]. Anti-TNF biologics have proved to be very effective in the induction and maintenance of remission in patients who do not respond to conventional immunosuppressive therapy [2]. Presently, there are five TNFi approved by EMA and FDA: adalimumab (ADAL); golimumab (another fully human IgG1 monoclonal antibody); infliximab (IFX) (a chimeric IgG1 monoclonal antibody); certolizumab (a PEGylated Fab fragment of a humanized anti-TNF monoclonal antibody); and etanercept (a fusion protein between a human IgG1 Fc-tail and human TNF-receptor type 2/TNFR2).

Although TNFis have significantly improved treatment outcomes for chronic inflammatory diseases, some patients have had to discontinue treatment due to primary non-response, secondary treatment failure, or adverse reactions. Primary non-response can occur in about 1/3 of patients, and up to 60% of initial responders lose response over time, defined as a need for dose escalation and drug discontinuation rates. A high suboptimal response rate or lack of response to first-line therapy with TNFi can be caused by non-immune related factors that affect drug clearance, such as high body mass index (BMI) or high disease burden. However, one of the main reasons for the loss of therapeutic efficacy of TNFis (secondary failure) is immunogenicity, namely the development of anti-drug antibodies (ADAs), especially drug-neutralizing ADAs against chimeric, humanized, or fully human TNFi [3].

ADAs have been linked to subtherapeutic serum drug levels by increasing the clearance and reducing the bioavailability of the TNFi, thus determining loss of clinical response. Moreover, ADAs have been linked to infusion-related adverse events, including hypersensitivity reactions [4]. Close monitoring of patients’ TNFi trough levels (i.e., therapeutic drug monitoring or TDM) has been proven to be a valuable tool to aid therapy optimization, as well as being a cost-effective strategy [5,6].

To address loss of response, several TDM algorithms have been proposed, which commonly involve assessment of drug trough concentrations and ADA levels. Changing the dosing regimen or dose intensity is often the first choice for treatment optimization. Other options, such as adding immunosuppressive co-medication, switching to a different TNFi (cycling strategy), or different classes of immunomodulatory drugs, can be potentially considered, especially in patients who develop ADAs.

Reliable assays measuring TNFi with accuracy and precision are a prerequisite for successful TDM. Several methods have been described to measure ADA levels, including different formats of ELISA, radioimmunoassays, liquid-chromatography-based homogeneous mobility shift assay (HMSA), or chemiluminescence immunoassays (CLIA). These methods use different designs and detection antibodies, and are differently prone to interference by TNFi, rheumatoid factors (RFs), or other interference factors [7]. Currently in clinical practice, ELISA tests represent the most commonly used type of assay for TDM in TNFi-treated patients. However, these assays often require several hours to complete without random access flexibility functioning. This may limit the effectiveness of TDM by significantly delaying dose adjustment in the subsequent drug administration, or delaying changes in treatment strategy.

This study aimed to evaluate the performance of the latest generation chemiluminescence random-access immunoassay analyzer i-TRACK^10®^ (Theradiag). Its performance was compared with the currently used Lisa-Tracker^®^ ELISA assays, which were performed manually or automatically using the DS2 ELISA processing instrument (Theradiag). These assays were used to monitor trough levels of IFX or ADAL, and ADA concentrations in TNFi-treated patients.

## 2. Results

### 2.1. Sample and Assay Characteristics

The key features of the analytical systems used in the study are summarized in Table 1. The main difference was the extended measurement range demonstrated by the i-TRACK^10®^ analyzer, both for serum drug concentration and ADA quantification. There was no difference for the coated antigen used (human recombinant TNF for IFX and ADAL assays, and the respective drugs for the aIFX and aADAL assays). No interference with hemolysis, bilirubin, triglyceride, rheumatoid factors, or biotin in the sample matrix has been reported for the assays analyzed in the study. Importantly, no significant cross-reactivity has been described with other TNFis, other biological classes, or molecules within the same disease spectrum. In the absence of prior dissociation of the immune complexes formed by the drugs and ADAs, the assays were considered drug-sensitive (reduced sensitivity to detect ADAs in the presence of the free drug).

### 2.2. Imprecision

The overall mean concentrations for each parameter (IFX, ADAL, and ADAs) and the imprecision values are represented in Table 2 and Supplementary Figure S1. Intra- and inter-run imprecisions were acceptable for all the assays (CV ranging from 1.8% to 16.1%) according to the current FDA guidelines [8]. Indeed, 20% is defined as the limit of acceptable CV% for precision and accuracy. Intra- and inter-run variations were significantly different for all the assays, except IFX and aIFX measurement at the high level solely for the B laboratory. The highest value was obtained for the inter-run imprecision of IFX in laboratory B. Intra-run CV ranged from 1.8% to 11.3%, and the mean CV was 8.6% for drugs and 2.7% for ADA quantification. Inter-run CV ranged from 7.3% to 16.1%, and the mean CV was 11.2% for drugs and 7.9% for ADA quantification.

### 2.3. Comparison of IFX and ADAL trough Levels

#### 2.3.1. Comparison between i-TRACK^10®^and Lisa Tracker^®^ for IFX and ADAL Quantification

The results for each assay were distributed into three categories: sub-therapeutic (<3 µg/mL for IFX and <5 µg/mL for ADAL), optimal range (3–7 µg/mL for IFX and 5–8 µg/mL for ADAL), and high range (>7 µg/mL for IFX and >8 µg/mL for ADAL) (Table 3). The kappa values were satisfactory according to the recommendations of Landis et al. [9], and slightly better between i-TRACK^10®^ and DS2^®^ LT^®^ (kappa value = 0.97) than manual LT^®^ (kappa value = 0.81) for IFX quantification. A change of classification for two samples was observed between i-TRACK^10®^ and manual LT^®^. Results were considered in the high range for i-TRACK^10®^ and in the optimal range for manual LT^®^. Change of classification for one sample was observed between the i-TRACK^10®^ and DS2 LT^®^ results. The sample was considered in the sub-therapeutic range by i-TRACK^10®^ and in the optimal range by DS2^®^ LT^®^.

For ADAL quantification, the kappa value was slightly lower but was satisfactory and similar to the two LT^®^ methods (kappa value = 0.7). Comparing the results obtained with i-TRACK^10®^ and manual LT^®^, a change of classification was observed for three samples: two that were in the optimal range according to i-TRACK^10®^ and were found to be in the sub-therapeutic range by the manual LT^®^ assay, and one sample that was in the high range with i-TRACK^10®^ and in the optimal range with the manual LT^®^ assay. The comparison between i-TRACK^10®^ and DS2^®^ LT^®^ showed ten particular discrepancies: six samples were in the sub-therapeutic range with i-TRACK^10®^ and in the optimal range with DS2^®^ LT^®^, two samples that were in the high range level according to i-TRACK^10®^ were found by DS2^®^ LT^®^ to be in the optimal range, and two samples were in the optimal range with i-TRACK^10®^ and in the high range with DS2^®^ LT^®^.

#### 2.3.2. Correlation between i-TRACK^10®^and Lisa-Tracker^®^ Assay Results

**Figure 1:**
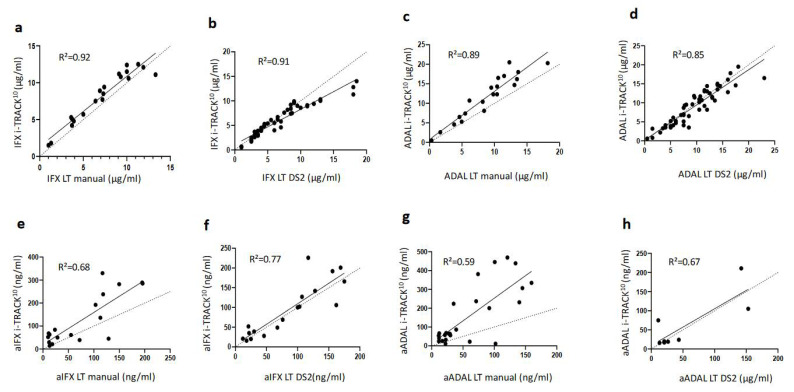
**(a**–**h)** Comparison between IFX, ADAL, aIFX, and aADAL data obtained using i-TRACK^10®^ and Lisa-Tracker^®^ (LT) assay performed manually or with the DS2 instrument. R² values are shown for all linear correlations (**a**–**h**).

**Figure 2:**
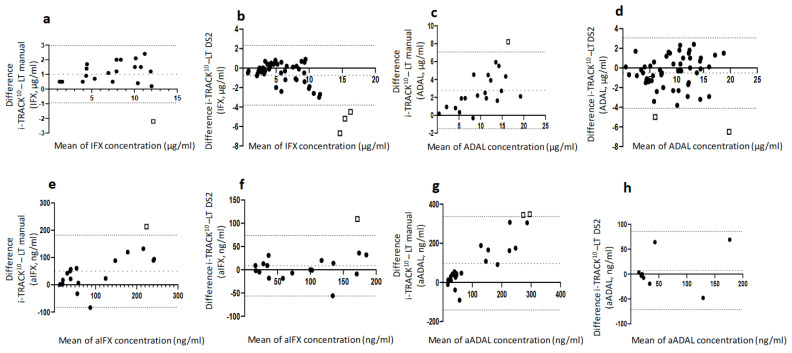
**Bland–Altman plots to compare different assays:** (**a**,**b**) Comparison of IFX level between i-TRACK^10**®**^ and Lisa tracker**^®^** (LT) assays; (**c**,**d**) comparison of ADAL level between i-TRACK^10**®**^ and Lisa-Tracker^®^ assays; (**e**,**f**) comparison of aIFX level between i-TRACK^10**®**^ and LT^®^ assays; (**g**,**h**) comparison of aADAL level between i-TRACK^10®^ and LT^®^ assays. The difference between the two measurements (µg/mL for drugs and ng/mL for ADAs) is plotted on the y-axis, and the average of the two measurements on the x-axis. Dashed lines represent the bias and the 95% limits of agreement for each comparison.

Linear regressions and Bland–Altman plots for each pair of assays are represented in Figure 1a–d and Figure 2a–d, respectively. Excellent linear correlations, between 0.85 and 0.92, were obtained for each pair of assays. Globally, for all comparisons, the difference between assays was slightly more prominent for the highest values of IFX and ADAL levels, but remained acceptable. Analysis of systematic bias from the Bland–Altman plots indicated that IFX levels were 17% higher on average in the i-TRACK^10®^ group compared to data obtained from the manual LT^®^ (95% CI: −9.1%, 43.6%), with one point outside the 95% CI (−0.9, 3; bias 1, Figure 2a). The results for the IFX performed with i-TRACK^10®^ and DS2^®^ LT^®^ were closer to similarity. Bland–Altman plot analysis showed that recorded IFX levels were on average 10% lower using i-TRACK^10®^ (95% CI: −46.7%, 26.5%), and the values were aggregated, especially within the lower range (under 10 µg/mL). There were three points outside the 95% CI (−3.7, 2.3; bias −0.7), which were connected to results within a higher range of the values (Figure 2b). Average ADAL levels were 28% higher according to i-TRACK^10®^ compared with results obtained using manual LT^®^ (95% CI: −2.2%, 58.1%), with one point outside the 95% CI (−1.5, 7, bias 2.8, Figure 2c). The comparison of ADAL levels between i-TRACK^10®^ and DS2^®^ LT^®^ was similar, with the results of ADAL levels on average 8% lower using i-TRACK^10®^ (95% CI: −55.7%, 40.2%) compared with DS2^®^ LT^®^, and with two points lying outside the 95% CI range (−4.1, 3.1; bias −0.5), Figure 2d.

### 2.4. Comparison of Anti-IFX and Anti-ADAL ADA Testing

#### 2.4.1. Good Agreement between i-TRACK^10®^and Lisa-Tracker^®^ Measurements of aIFX and aADAL

To render the results clinically relevant for patient-centered outcomes, a value of 100 ng/mL of ADAs was chosen to distinguish between low or moderate levels—which could represent low affinity or transient ADAs, and high ADA values which would be associated with a decrease of circulating TNFis and a loss of treatment efficacy. For the drug assay, the agreement between i-TRACK^10®^ and DS2^®^ LT^®^ for aIFX and aADAL (kappa values = 1) was better than with the manual LT^®^ assay (kappa values = 0.89 and 0.67 for aIFX and aADAL, respectively) (Table 3). Nevertheless, the results obtained with i-TRACK^10®^ agreed well with those obtained by the two LT assays, as previously reported by Landis et al. [8]. Concerning aIFX, a change of classification for only one sample was observed between i-TRACK^10®^ and manual LT^®^. The result was considered within the low/middle value range according to i-TRACK^10®^, and within the high range by manual LT^®^. Concerning aADAL, a change of classification was observed for six samples: four samples were considered within the low/middle range values by manual LT^®^ and within the higher range values by i-TRACK^10®^, and two samples were within the high range values according to manual LT^®^ and within the lower range values for i-TRACK^10®^.

#### 2.4.2. Correlations between i-TRACK^10®^and Lisa-Tracker^®^ Assays for ADA Detection and Measurements

Linear correlations between ADA values obtained with different assays were between 0.59 and 0.77. For drugs, the differences were most significant within the highest ranges of aIFX and aADAL values, but remained acceptable (Figure 1e–h). Analysis of systematic bias from Bland–Altman plots indicated that aIFX levels were 50.7% higher on average when using the i-TRACK^10®^ analyzer compared with the manual LT^®^ assay (95% CI: −74%, 176.3%), with one result outside the 95% CI range (−82.3, 182.3; bias 50, Figure 2e). For drug levels, the observed differences were lower when comparing values obtained from i-TRACK^10®^ and DS2^®^LT^®^ analyzers. Analysis of systematic bias from the Bland–Altman plots indicated that aIFX levels were on average 9.3% higher using i-TRACK^10®^ compared with DS2^®^LT^®^ (95% CI: −61.8%, 80.5%), with one point outside the 95% CI range (−56.3, 73.7; bias 8.7, Figure 2f). The average aADAL levels were 67.8% higher using i-TRACK^10®^ compared with the manual LT^®^ assay (95% CI: −77.3%, 212.8%), with two results lying outside the 95% CI range (−140.6, 335.8; bias 97.6, Figure 2g). There were only eight points of comparison for aADAL values between i-TRACK^10®^ and DS2^®^LT^®^, where the observed trend included an equivalence of results between the two analyzers with no point lying outside the 95% CI range (−71.3, 85.6; bias 7.1, Figure 2h).

### 2.5. Sample to Sample Carryover

The assessment of sample carryover phenomena did not reveal any cross-contamination risks with the i-TRACK^10®^ analyzer, because the carryover index calculated was always less than 1% between runs (the obtained values were 0.28%, 0.59%, 0.03%, and 0.26% for IFX, ADA, aIFX, and aADAL, respectively).

## 3. Discussion

This bi-centric study aimed to validate a new chemiluminescent immunoassay random-access analyzer i-TRACK^10®^ (Theradiag) to monitor treatment with IFX and ADAL, for detection and quantification of drug levels and ADAs. These two drugs were chosen because these treatments are frequently prescribed for many indications, notably in gastroenterology and rheumatology, and request for their dosage is very common in many labs, not only in university hospital laboratories. We consider that these results can be extrapolated to other molecules, for example, vedolizumab and ustekinumab.

Intra-run and inter-run comparisons were performed between i-TRACK^10®^ and standard manual or automated (DS2^®^) Lisa-Tracker^®^ assays (both from Theradiag), in addition to carryover phenomenon assessment. We found acceptable intra- and inter-run CV values for all the parameters tested, which were similar between the two labs. Our acceptance criterion was 20% CV as proposed by FDA guidelines [8]. The obtained CVs were consistent with our previous evaluations of manual and DS2^®^ Lisa-Tracker^®^ assays, which showed CV values between 5% for the minimal intra-run and 15% for the maximal inter-run results. As expected, the inter-run CVs were globally superior to those obtained in the intra-run assays, but there was no substantial difference concerning the imprecision of drug and ADA values and between the different ranges of the datasets. The previous i-TRACK^10®^ evaluation by the manufacturer showed an intra-run CV ranging from 1.6% to 8.1% (run of ten points) for IFX assay, and from 1.7% to 11.8% for the aIFX assay. For the inter-run assays (six independent runs), CV ranged from 2% to 6.7% for IFX and from 2.3 to 4.8% for aIFX [10].

The present study revealed an acceptable correlation between i-TRACK^10®^ and automated or manual LT^®^ assays, especially for lower concentration ranges, which represent the most interesting group of results because these can impact clinical decision-making. As shown in previous studies [11,12,13,14,15], the correlations were better for drugs than for their corresponding ADAs (R² ranged from 0.85 to 0.92 and from 0.59 to 0.77, respectively). The lowest correlation was observed between i-TRACK^10®^ and manual LT^®^ values for aADAL, with an R² value of 0.59. The results were often higher for i-TRACK^10®^ (on average 67.8% higher), with a Bland–Altman bias at 97.6. This overestimation encountered when using the i-TRACK^10®^ analyzer was not mentioned in the manufacturer’s internal evaluation report and is probably linked to the expanded measurement range.

We next tested whether the type of assay used could change the clinical interpretation and subsequent attitude, especially in patients with chronic inflammatory bowel diseases. With this aim, we classified our results into different concentration ranges based on therapeutic windows defined in several previous studies [16,17,18,19,20,21]. We found that different assays suggested a change of category for 4% of the IFX samples, 17% of the ADAL samples, 3% of the aIFX samples, and 11% of the aADAL samples. We based our interpretation on the cut-off used in clinical practice to define responder versus non-responder patients, although these cut-off values are better defined for drugs [16,17,20,21] than for ADAs. The kappa coefficient was globally acceptable for all the comparisons tested, according to the acceptance criterion of Landis et al. [9]. Nevertheless, the type of assay, in particular i-TRACK^10®^ versus manual LT^®^ assay, was found in our study to induce a change of interpretation for ADA values, namely with a trend towards overestimation of ADA levels using the i-TRACK^10®^ analyzer. It is worth noting that drug and ADA monitoring tests are not fully interchangeable, especially during the longitudinal follow-up of a given patient, as previously reported by Pérez et al. [22]. A common clinical recommendation is to use the same assay format for long-term follow-up. However, the observed differences in our study particularly concern values within ranges, thus they have less potential to affect clinical decision-making.

The i-TRACK^10®^ analyzer presents many advantages, mentioned in Table 4, particularly its expanded measurement ranges for ADAs with the highest point of the standard curve approximately 10-fold higher than in the LT^®^ kit. Another major advantage is the rapidity of access to results. ELISA techniques require significant and incompressible hands-on time and work by series, which significantly lengthens the time-to-result performance parameter. The i-TRACK^10®^ instrument has also benefits linked to standardized techniques, with very low imprecision and personal or instrumental errors.

Our study has some limitations, including the small number of samples tested, in particular in the aADAL group compared between DS2^®^ LT^®^ and i-TRACK^10®^, thereby limiting its overall strength somewhat. However, this explorative bi-centric study is the first to have been devoted to evaluating the i-TRACK^10®^ analyzer as a part of its future accreditation process.

## 4. Methods

### 4.1. Patients and Samples

Samples were obtained from patients treated with IFX and ADAL for whom drug levels and ADA concentrations were assessed as part of regular medical care, in two different laboratories in France, CHU Saint-Etienne (laboratory A) and Bicêtre Hospital at Le Kremlin-Bicêtre (laboratory B). Blood samples were collected immediately before TNFi administration. These were centrifuged, and the patients’ sera were stored at −20 °C and thawed only once before use. The remaining samples from the routine laboratory were selected and de-identified before use. Samples were used for the comparison assays (different samples from the two labs), and the intra-run assays for laboratory A. Quality controls (IMMUNO-TROL^®^, Theradiag, Croissy Beaubourg, France) were used for the intra-run assays in laboratory B, and for the inter-run assays in both labs. The quality controls (QC) were analyzed in the same manner as the patient samples, and were used at two concentrations: a low/medium level (<4 µg/mL for drugs and <100 ng/mL for ADAs) and a high level (>9 µg/mL for drugs and >100 ng/mL for ADAs). The number of samples used for all evaluations is presented in Supplementary Table S1. No ethical approval was required for the study, which was conducted for quality assurance purposes only (Code de Santé Publique Français article L1243-3 and L1121-1).

### 4.2. Methods

Three assays were compared for this study: Lisa-Tracker^®^ (LT) (Theradiag) with a manual ELISA procedure (manual LT) (laboratory A); or with an automated ELISA DS2^®^ analyzer (DS2 LT) (Dynex technologies, Chantilly, VA, USA) (laboratory B); and the i-TRACK^10®^ analyzer (laboratories A and B). The LT procedure was performed according to the manufacturer’s instructions and the protocol for the DS2 analyzer was identical to the manual one. The distribution and washing steps were automated for the DS2, and the optical density (OD) reading was performed using the spectrophotometer incorporated inside the analyzer.

### 4.3. Data Analysis

As recommended by the quality assurance guidelines, the intermediate intra-run and inter-run measurements of imprecision (CV, coefficient of variation (%)) of the i-TRACK^10®^ were evaluated for each of the parameters tested (IFX, ADAL, aIFX, aADAL). Intra-run and inter-run variances for each parameter in the same lab were compared using Welch’s *t*-test, and *p* < 0.05 was set as the level of significance.

For qualitative analysis, the agreement between results obtained with the i-TRACK^10®^ and the LT^®^ technology was assessed using Cohen’s kappa coefficient, considering the value of (a) as zero if there was no more agreement than could be expected by chance, or (b) as 1 if there was a perfect agreement.

Kappa results were interpreted as follows: values lower than 0.2 indicated a slight agreement, between 0.2 and 0.4 the agreement was considered as fair, between 0.4 and 0.6 as moderate, between 0.6 and 0.8 as substantial, and values greater than 0.8 indicated almost perfect agreement [9]. For quantitative comparison, linear regression was evaluated using an x–y plot, and the R² correlation coefficient was calculated. A value of 1 indicated perfect linear correlation, while a value of 0 translated to an absence of correlation. Bland–Altman plots were performed with GraphPad Prism version 8 (GraphPad Software, La Jolla, CA, USA), and the mean difference between i-TRACK^10®^ and other systems and the 95% limits of agreement were calculated. A *p*-Value < 0.05 was considered statistically significant.

Sample carryover effect was assessed, according to the general quality assurance protocol, by selection and analysis of two patient samples, one with high (H) and another with low (L) values of IFX, ADAL, aADAL, and aIFX. The same H sample was analyzed in triplicate followed by three analyses of the same L sample. Carryover was calculated for each analyte as follows: ((L1-L3)/(H3-L3)) × 100. A value of carryover below 1% was considered insignificant.

## 5. Conclusions

We performed a complete evaluation of a new random-access analyzer i-TRACK10^®^ for the monitoring of TNFi biologics. Despite good overall performances and correlations between i-TRACK^10®^ and Lisa-Tracker assays^®^, we recommend using the same assay format for long-term follow-up of patients treated by TNFi biologics.

## Figures and Tables

**Table 1:** Assay characteristics.

	Antigen		Method	Measurement Range				Interference
	IFX/ADAL	aIFX/aADAL ADAs		IFX	ADAL	aIFX	aADAL	
**Lisa Tracker^®^** **assays**	Human recombinant TNF	Infliximab Adalimumab	Manual or automated (DS2) ELISA	0.3–20 µg/mL		10–200 ng/mL	10–160 ng/mL	No influence onhaemolysis, bilirubin, triglyceride, RF. No cross-reaction with other anti-TNF molecules nor with rituximab.
**i-TRACK^10®^** **assay**	Human recombinant TNF	Infliximab Adalimumab	CLIA	0.3–24 µg/mL	0.5–24 µg/mL	10–2000 ng/mL		No influence of haemolysis (2 mg/mL), bilirubin (0.2 mg/mL), triglyceride (10 mg/mL), RF (1000 UI/mL), biotin (2000 ng/mL). No cross-reactivity with other anti-TNF biologics, ustekinumab/vedolizumab, nor with aADAL ADAs (for IFX), nor with aIFX ADAs (for ADAL).

CLIA: ChemiLuminescence ImmunoAssay; ELISA: Enzyme-Linked ImmunoAssay; IFX: infliximab; ADAL: adalimumab; aIFX: anti-infliximab antibodies; aADAL: anti-adalimumab antibodies; ADAs: anti-drug antibodies; RF: rheumatoid factors. Source: Technical material for Lisa Tracker^®^ and i-TRACK^10®^ instruments.

**Table 2:** Imprecisions of the i-TRACK^10^^®^ instrument with the use of sample patients or QC (low/medium and high values). For each analyte, the level of concentration used for the assay and the obtained CVs are mentioned. ND: Not determinated.

	Laboratory A		Laboratory B	
	*Low or Medium*	*High*	*Low or Medium*	*High*
Mean IFX—µg/mL (Intra-run—CV%)	1.6 (8.2)	9.1 (11.3)	2.1 (5.8)	10.7 (8.3)
Mean IFX—µg/mL (Inter-run—CV%)	2.3 (11.7)	11.7 (7.3)	2.3 (16.1)	10.3 (10)
Mean ADAL—µg/mL (Intra-run—CV%)	1 (8.1)	13.3 (10.3)	ND	ND
Mean ADAL—µg/mL (Inter-run—CV%)	3.7 (9.3)	12.5 (12.8)		
Mean aIFX ADAs—ng/mL (Intra-run—CV%)	30 (3.7)	102 (3.7)	56 (1.8)	586 (2.6)
Mean aIFX ADAs—ng/mL (Inter-run—CV%)	60 (6.3)	603 (4.8)	61 (13.5)	594 (10.2)
Mean aADAL ADAs—ng/mL (Intra-run—CV%)	37 (1.8)	212 (2.2)	ND	ND
Mean aADAL ADAs—ng/mL (Inter-run—CV%)	53 (4.1)	505 (8.6)		

**Table 3:** Data agreement between i-TRACK^10^^®^ and manual/DS2 Lisa Tracker^®^ (LT) values for IFX, ADAL, aIFX, and aADAL quantifications, according to therapeutic window stratification. The number of samples that changed classification is indicated in bold and underlined.

IFX	I-TRACK^10^			
Manual LT *DS2 LT*	<3 µg/mL	3–7 µg/mL	>7 µg/mL	TOTAL
**<3 µg/mL**	2 *7*	0 *0*	0 *0*	2 *7*
**3–7 µg/mL**	0 ***1***	5 *24*	**2** *0*	7 *25*
**>7 µg/mL**	0 *0*	0 *0*	11 *18*	11 *18*
**TOTAL**	2 *8*	5 *24*	13 *18*	20 *50*
**Kappa value**	**0.81** ***0.97***			
**ADAL**	**I-TRACK^10^**			
**Manual LT** ***DS2 LT***	**<5 µg/mL**	**5–8 µg/mL**	**>8 µg/mL**	**TOTAL**
**<5 µg/mL**	3 *7*	**2** *0*	0 *0*	5 *7*
**5–8 µg/mL**	0 ***6***	1 *8*	**1** ***2***	2 *16*
**>8 µg/mL**	0 *0*	0 ***2***	13 *30*	13 *32*
**TOTAL**	3 *13*	3 *10*	14 *32*	20 *55*
**Kappa value**	**0.7** ***0.66***			
**aIFX **	**I-TRACK^10^**			
**Manual LT** ***DS2 LT***	**<100 ng/mL**	**≥100 ng/mL**		**TOTAL**
**<100 ng/mL**	10 *9*	0 *0*		10 *9*
**≥100 ng/mL**	**1** *0*	7 *9*		8 *9*
**TOTAL**	11 *9*	7 *9*		18 *18*
**Kappa value**	**0.89** ***1***			
**aADAL**	**I-TRACK^10^**			
**Manual LT** ***DS2 LT***	**<100 ng/mL**	**≥100 ng/mL**		**TOTAL**
**<100 ng/mL**	35 *4*	**4** *0*		39 *4*
**≥100 ng/mL**	**2** *0*	9 *2*		11 *2*
**TOTAL**	37 *4*	13 *2*		50 *6*
**Kappa value**	**0.67** ***1***			

**Table 4:** List of the advantages and disadvantages of the manual and automatic Lisa-Tracker^®^ and I-TRACK^10^ systems.

	Manual Lisa Tracker^®^	DS2 L Tracker^®^ (Specific to the Automated Process)	I-TRACK^10^^®^
Advantages	Reliable and robust test to quantify drugs and anti-drug antibodies Precision, sensibility and specificity of the dosage Many molecules and their specific antibodies are available	No risk of human error affecting sample dilution and distribution	Random-access instrument: decreased time to access the result for clinicians (about 35 min to obtain the first result) Standardization thanks to the automated process Reducing the chance of data input errors with automated transmission to the informatics lab system Expansion of the measuring range, especially for ADA, useful for therapeutic de-escalation Precision, sensibility and specificity of the dosage Possibility of dosing multiple drugs and anti-drugs antibodies in the same round Cost
Disadvantages	Need to work in series Unavoidable risk of technical issues (manual dilution of samples, distribution, results entry) Incompressible technical time linked to manual steps Increased time-to-result (about 120 min to obtain the first result)	Risk of machine failure	Risk of machine failure Delay of the accessibility to the dosage in the instrument (for molecules infrequently prescribed)

## Data Availability

Datas are available upon request.

## Supplementary Materials

The following supporting information can be downloaded at: https://www.mdpi.com/article/10.3390/ijms23179561/s1.

## Abbreviations

ADAL: adalimumab; aADAL: anti-adalimumab antibodies; CLIA: ChemiLuminescence ImmunoAssay; CV: coefficient of variation; IFX: infliximab; aIFX: anti-infliximab antibodies; LT: Lisa-Tracker^®^ ELISA kit; LLOQ: Low Limit of Quantification; TNF: Tumor Necrosis Factor, TNFi: TNF inhibitors.
