# Supplementary material for: Validation Study of a New Random-Access Chemiluminescence Immunoassay Analyzer i-TRACK10® to Monitor Infliximab and Adalimumab Serum trough Levels and Anti-Drug Antibodies
Source: Int J Mol Sci. 2022 Aug 24;23(17):9561. doi: 10.3390/ijms23179561 (PMC9455629; doi:10.3390/ijms23179561)
Supplement: Supplementary file 1 [file ijms-23-09561-s001.zip › ijms-1848373-supplementary.pdf]

Validation study of a new random-access chemiluminescence immunoassay analyzer i-TRACK10® to monitor infliximab and adalimumab serum trough levels and anti-drug antibodies

Anne-Emmanuelle BERGER1,2,3, Aude GLEIZES4,5,6, Louis WAECKEL1,2,3, Xavier ROBLIN1,2,7, Roman KRZYSIEK4,8, Salima HACEIN-BEY-ABINA4,5, Alessandra SORIANO9 and Stephane PAUL1,2,3.[1] Affiliation 1; e-mail@e-mail.com

[1]CIRI – Centre International de Recherche en Infectiologie, Team GIMAP (Saint-Etienne), Université Claude Bernard Lyon 1, Inserm, U1111, CNRS, UMR5308, ENS Lyon, UJM, F69007 Lyon, France.

[2]CIC Inserm 1408 Vaccinology, F42023 Saint-Etienne, France.

[3]Department of Immunology, CIC1408, GIMAP U1111/UMR5308 INSERM-UJM-UCBL-ENS de Lyon-CNRS, University Hospital of Saint-Etienne, Saint-Etienne, France

[4]Immunology Laboratory, Groupe Hospitalier Universitaire Paris-Saclay, Hôpital Bicêtre, Assistance Publique-Hôpitaux de Paris,Le-Kremlin-Bicêtre, France

[5]Université de Paris, CNRS, INSERM, UTCBS, Unité des technologies Chimiques et Biologiques pour la Santé,Paris, France

[6]Université Paris-Saclay, UFR de Pharmacie, Châtenay-Malabry, France.

[7]Gastroenterology department, CHU Saint-Etienne, Saint-Etienne, France.

[8] Université Paris-Saclay, UMR-996 INSERM Inflammation, Microbiome and Immunosurveillance, Faculté de Médecine, Clamart, France

[9] Gastroenterolgoy Division and IBD Center, Internal Medicine Department, Azienda Unità Sanitaria Locale – IRCCS, Reggio Emilia, Italy

* Correspondence: **author**

Dr. Anne-Emmanuelle BERGER; a.emmanuelle.berger@chu-st-etienne.fr

Dr. Alessandra SORIANO; alessandra.soriano@ausl.re.it

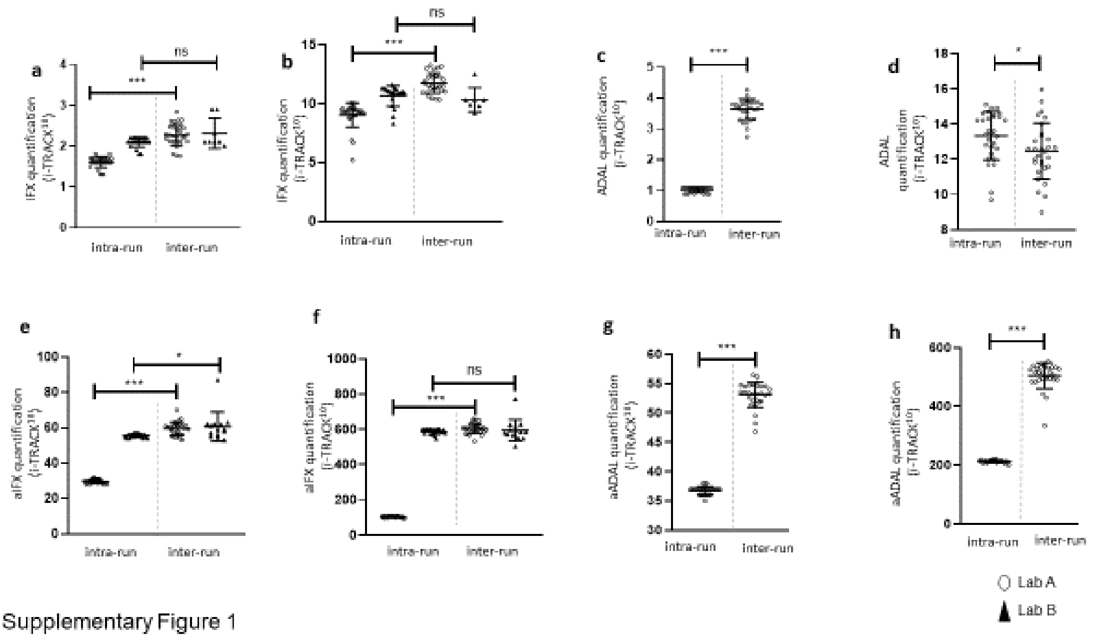

Supplementary Figure 1

Supplementary Figure S1. i-TRACK10® intra-run and inter-run variations for IFX (µg/ml; a-b), ADAL (µg/ml; c-d), aIFX ADAs (ng/ml; e-f) and aADAL ADAs (ng/ml; g-h) assays in two laboratories for low (a, c, e, g) and high (b, d, f, h) values. Means and SD are represented by horizontal bars and statistical significance between intra-run and inter-run assays is shown (Welch T test and comparison of variances, NS: non statistically significant, *** p<0.0001). *No data could be obtained for ADAL and aADAL for Lab B.*

Supplementary Table S1. Number of samples used in two laboratories (Lab A and Lab B) for intra/inter-runs and comparison assays (i-TRACK^{10}® versus manual and DS2 Lisa Tracker® (LT)).

**Number of samples (Patients' sera or Quality Controls)**	**IFX**	**ADAL**	**aIFX ADAs**	**aADAL ADAs**
**Intra-run assay**	***Lab A* 30**<br>***Lab B* 20**	***Lab A* 30**	***Lab A* 30**<br>***Lab B* 20**	***Lab A* 30**
**Inter-run assay**	***Lab A* 30**<br>***Lab B* 20**	***Lab A* 30**	***Lab A* 30**<br>***Lab B* 14**	***Lab A* 30**
**I-TRACK^{10} versus manual LT**	**20**	**20**	**18**	**50**
**I-TRACK^{10} versus DS2 LT**	**50**	**55**	**18**	**6**
